# BacExplorer: an integrated platform for *de novo* bacterial genome annotation

**DOI:** 10.1093/bioadv/vbaf281

**Published:** 2025-11-09

**Authors:** Grete Francesca Privitera, Adriana Antonella Cannata, Floriana Campanile, Salvatore Alaimo, Dafne Bongiorno, Alfredo Pulvirenti

**Affiliations:** Department of Clinical and Experimental Medicine, Bioinformatic Unit, University of Catania, Catania 95123, Italy; Department of Mathematics and Computer Science, University of Catania, Catania 95123, Italy; Department of Biotechnology and Biomedical Science, Microbiology Section, University of Catania, Catania 95123, Italy; Department of Clinical and Experimental Medicine, Bioinformatic Unit, University of Catania, Catania 95123, Italy; Department of Biotechnology and Biomedical Science, Microbiology Section, University of Catania, Catania 95123, Italy; Department of Clinical and Experimental Medicine, Bioinformatic Unit, University of Catania, Catania 95123, Italy

## Abstract

**Motivation:**

High-throughput sequencing (HTS) has become an integral part of routine analysis for microbiologists. The process of sequencing dozens of samples generates vast amounts of data that cannot be annotated manually. To address this challenge, numerous tools for bacterial genome analysis have been developed over the years. Using freely available databases, these tools enable users to significantly accelerate their analyses. However, many of these tools require advanced computer science expertise to operate effectively.

**Results:**

To overcome this limitation, we developed BacExplorer. Featuring a user-friendly interface, a locally installable application, and an interactive HTML report, BacExplorer empowers users of all skill levels to perform their own analyses with ease and efficiency.

**Availability and implementation:**

BacExplorer is available at: https://github.com/knowmics-lab/BacExplorer

## 1 Introduction

Pathogen surveillance using high-throughput DNA sequencing (HTS) is becoming more popular day by day. The field of infectious diseases is experiencing significant transformation due to the emergence of affordable whole genome sequencing (WGS) technologies. The next generation sequence (NGS) analysis is rapidly becoming a standard practice, revolutionizing laboratory protocols. WGS offers a more accurate and efficient approach by directly analyzing the bacterial genome, facilitating precise species identification, strain typing, and prediction of drug resistance and virulence gene profiles (Fricke and Rasko 2014). The advantages and transformative potential of NGS, along with the essential role of bioinformatics, are proving invaluable in utilizing WGS data for better patient care and public health. These advances provide extraordinary insights into microbial diversity and evolution, enable highly accurate prediction of antimicrobial resistance (AMR) genes and mutations, and support timely and informed treatment decisions. Furthermore, they improve the epidemiological surveillance of bacterial infections, including the detection of emerging pathogens and tracking the spread of AMR (Allard *et al.* 2019, Su *et al.* 2019).

Bacterial infections pose a significant global health burden, accounting for millions of deaths annually. Laboratory diagnosis traditionally relies on culture-based methods, considered the “gold standard,” but these can be time-consuming and ineffective for some pathogens; conversely, molecular methods offer significant advantages over traditional methods. They are generally more sensitive, faster, and applicable to both culturable and non-culturable organisms (Schmitz *et al.* 2022). A wide range of tools is available, with new ones continuously being developed to enhance the accuracy and efficiency of WGS in microbial analysis. These tools enable the detailed characterization of microbial features such as the “resistome”—the collection of AMR genes and their precursors within a bacterial population—and the “virulome,” which represents the virulence genes responsible for a microorganism’s pathogenic potential. Furthermore, they support the comprehensive study of other microbial traits critical for advancing research, guiding treatment decisions, and bolstering public health efforts (Singh *et al.* 2022).

Among these tools we can mention: Bactopia (Petit and Read 2020), Tormes (Quijada *et al.* 2019), Campype (Ortega-Sanz *et al.* 2023), Bacpipe (Xavier *et al.* 2020), Patric (Gillespie *et al.* 2011), Asa3P (Schwengers *et al.* 2020), and Rmap (Sserwadda and Mboowa 2021). All of them present both strength and weakness. For instance, while Bactopia includes numerous tools for bacterial analysis and is among the most comprehensive options available, it lacks an easily accessible report and a user-friendly interface that would allow microbiologists to use it independently. Additionally, most tools, except for Patric, require installation via the command line, which poses significant usability challenges for those without a background in computer science. All these tools are free; however, it is possible to find also easy-to-use software sold by companies. For instance, QUIAGEN CLC https://digitalinsights.qiagen.com/products-overview/discovery-insights-portfolio/analysis-and-visualization/qiagen-clc-genomics-workbench permits to the user to analyze raw fastq data giving as output AMR and virulence data, ridom SeqSphere (https://www.ridom.de/seqsphere/) it is usable trough an app easy to install which permits to analyze raw data from Illumina, IonTorrent, ONT and PacBio selecting the pipeline needed by the user (e.g. cgMLST). Moreover, illumina basespace (https://emea.illumina.com/areas-of-interest/microbiology/microbial-sequencing-methods/microbial-whole-genome-sequencing.html) analyzes directly the raw data from sequencing machines (e.g. MiSeq) giving as an output the sequence type (ST), the AMR associated genes, the virulence factor, and plasmid replicons.

In this paper, we present BacExplorer, a tool developed in Snakemake (Köster and Rahmann 2012) with an Electron (https://github.com/electron/electron) user interface that allows to analyze raw bacteria genomes both in fastq and in fasta format. The software is open source and gives as output an HTML report.

## 2 Methods

**Figure 1.**
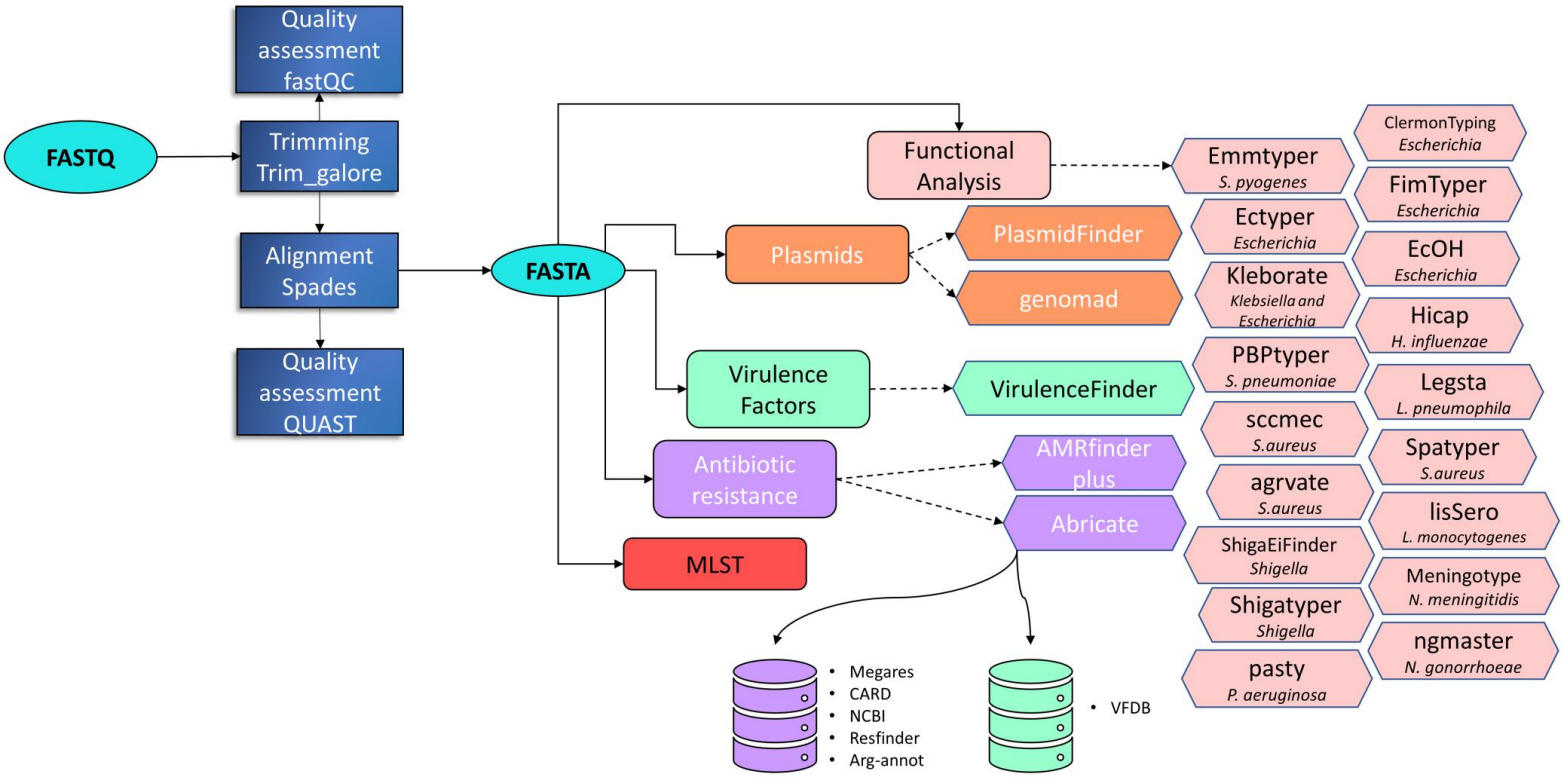
BacExplorer workflow.

The analysis pipeline implemented in BacExplorer (Fig. 1) is encapsulated within a Snakemake workflow. This Python-based workflow management system enables reproducible and scalable execution of bioinformatics tasks but is optimized for Linux environments. To ensure cross-platform compatibility and seamless deployment, we built a Docker container that includes Snakemake and all necessary dependencies (Merkel 2014).

To further improve usability, we developed a cross-platform desktop application with a user-friendly Graphical User Interface (GUI), built using the Electron framework, featuring a React front-end and Bootstrap styling. On first use, the application guides the user through the environment setup process. After manually verifying the presence of Docker on the user’s machine, the remainder of the setup is fully automated, including: (i) pulling the Docker image from Docker Hub, (ii) downloading required databases and external resources, and (iii) creating the Docker container.

Once the environment is configured, users can access the Analysis page to customize parameters for their run. This includes specifying the input folder, selecting the genus and species of the organisms to analyze, choosing the input file format (.fasta or .fastq), and launching the Snakemake workflow. Upon successful completion, users are redirected to a Report page, where the final output, generated via RMarkdown, is presented in a clear and interactive format, offering an accessible visualization of the analysis results.

### 2.1 Employed programs

The BacExplorer pipeline begins with quality control, genome cleaning and alignment, followed by annotation. The initial annotation focuses on AMR and virulence genes, progressing to species-specific analyses, such as *emm*-typing in *Streptococcus pyogenes*.

As shown in Table 1, BacExplorer integrates multiple software and databases to perform its analyses. These tools are organized based on their specific functions, ensuring a streamlined and efficient workflow.

**Table 1.** Summary of the employed software and databases of BacExplorer.

Organism	Resources	Function	Version
All	fastQC	Raw Reads quality control	0.12.1
All	Quast	Genome Quality Assessment	5.3.0
All	trimgalore	Trimming	0.6.10
All	SPAdes	*De novo* genome assembly	4.0.0
All	kraken2	Taxonomy identification	2.1.3
All	mlst	Scan contig files against traditional PubMLST typing schemes	2.23.0
All	ncbi-amrfinderplus	Antimicrobial resistance gene and virulence factor identification	3.12.8
All	ABRicate	Antimicrobial resistance gene and virulence factor identification	1.0.1
All	NCBI	Antimicrobial resistance database	15/12/2024
All	CARD	Antimicrobial resistance database	3.3.0
All	Megares	Antimicrobial resistance database	v2
All	ResFinder	Antimicrobial resistance database	22/03/2024
All	ARG-ANNOT	Antimicrobial resistance gene database	V6
All	VirulenceFinder	Virulence factor identification	2.0.4
All	VFDB	Virulence factor database	06/12/2024
All	PlasmidFinder	Plasmid identification	2.2.0
All	geNomad	Identification of mobile genetic elements	1.11.0
*S.aureus*	spaTyper	Spa type identification	0.3.3
	agrvate	agr locus typing	1.0.2
	sscmec	typing SCCmec cassettes	1.2.0
*Klebsiella spp.*	Kleborate	Screen Klebsiella genome assembly	3.1.2
*Escherichia spp.*	ecoh	E.coli serotyping	2019
	ecoli vf	Virulence factor database	2017
	FimTyper	FimH type identification	2017
	ClermonTyping	Escherichia strain phylotyping	24.02
	ECTyper	*E. coli* serotyping	0.8.1
*L. pneumophila*	Legsta	Sequence based typing	0.5.1
*L. monocytogenes*	LisSero	Serotype prediction	0.4.9
*S. pyogenes*	emmtyper	Automatic emm-typing of *S. pyogenes*	0.2.0
*S. pneumoniae*	pbptyper	Penicillin binding protein typer	2.0.0
*Shigella spp.*	ShigEifinder	Identify differentiate Shigella/EIEC and Serotype	1.3.5
	ShigaTyper	Shigella serotype prediction	2.0.5
*P. aeruginosa*	pasty	Serogrouping of *Pseudomonas aeruginosa* isolates	2.2.1
*H. influenzae*	hicap	cap locus identification	1.0.4
*Neisseria spp.*	ngmaster	*In silico* multi-antigen sequence typing for *Neisseria gonorrhoeae*	0.5.8
	meningotype	Typing of *Neisseria meningitidis*	0.8.5

- Quality assessment of raw samples using fastQC (https://www.bioinformatics.babraham.ac.uk/projects/fastqc/)and of assembled genome with QUAST (Gurevich *et al.* 2013);
- Cleaning of raw samples and alignment using trimgalore (Martin 2011) (https://github.com/FelixKrueger/TrimGalore) and SPAdes (Prjibelski *et al.* 2020);
- Taxonomy assignment with Kraken2 (Wood *et al.* 2019);
- Molecular Typing via multi-locus sequence typing (MLST) analysis (Jolley and Maiden 2010, Seemann 2022);
- Antibiotic resistance genes and virulence factors annotation using various software tools: (i) AMRfinder Plus (Feldgarden *et al.* 2021), a tool from NCBI that identifies both antibiotic resistance and virulence genes, applicable to unknown or specific organisms for more precise analysis; (ii) Abricate (https://github.com/tseemann/abricate), a software that searches for antibiotic resistance and virulence genes across multiple databases, including NCBI (Feldgarden *et al.* 2019), Megares (Doster *et al.* 2020), VFDB (Chen *et al.* 2016), Arg-annot (Gupta *et al.* 2014), and CARD (Jia *et al.* 2017); (iii) VirulenceFinder (Joensen *et al.* 2014), a tool specialized in the detection of virulence genes.
- Plasmid analysis using plasmidfinder (Carattoli *et al.* 2014) and geNomad (Camargo *et al.* 2024)
- Screening of *Klebsiella* spp. and *Escherichia* spp. genomes with Kleborate (Lam *et al.* 2021).
- *emm*-typing of *S. pyogenes* using Emmtyper (Frost *et al.* 2020).
- Multiple analysis on *Staphylococcus aureus*:
  - *spa* typing with SpaTyper (https://github.com/HCGB-IGTP/spaTyper)
  - *agr* locus typing and identification of variants in the *agr* operon using Agrvate (Raghuram *et al.* 2022)
  - SCC*mec* typing with SCCmec (https://github.com/rpetit3/sccmec)
- Species-specific analysis on *Escherichia coli* using EcOH (Ingle *et al.* 2016), ecoli_vf (https://github.com/phac-nml/ecoli_vf/), FimtTyper (Roer *et al.* 2017) and ClermonTyping (Le Nagard 2018).
- Identification of cap locus serotype and structure in *Haemophilus influenzae* with hicap (https://github.com/scwatts/hicap)
- Serotype determination *Shigella* spp. with ShigaTyper (Wu *et al.* 2019) and ShigEifinder (Zhang *et al.* 2021)
- In silico typing of Penicillin Binding Proteins (PBP) for *Streptococcus pneumoniae* using pbptyper (Li *et al.* 2016) (https://github.com/rpetit3/pbptyper)
- Sequence based typing (SBT) of *Legionella pneumophila* with legsta (https://github.com/tseemann/legsta)
- Serogroup typing prediction for *Listeria monocytogenes* with LisSero (Doumith *et al.* 2004)
- Multi-antigen sequence typing for *Neisseria gonorrhoeae* with ngmaster (Kwong *et al.* 2016) and typing of *Neisseria meningitidis* with meningotype (https://github.com/MDU-PHL/meningotype)
- Serogrouping of *Pseudomonas aeruginosa* with pasty (https://github.com/rpetit3/pasty)

## 3 Results

**Figure 2.**
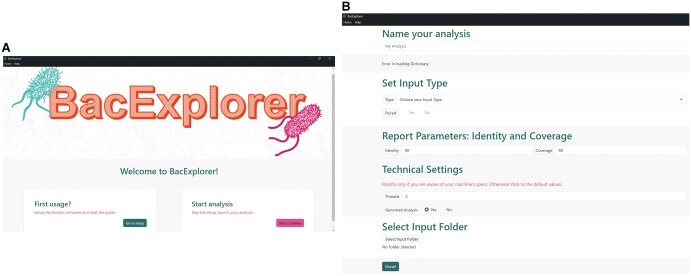
BacExplorer graphical user interface (GUI). A) Homepage of the BacExplorer application, providing options for initial environment setup (for first-time users) and for launching a new analysis. B) Analysis page with configurable parameters, including input file type (FASTA or FASTQ), coverage thresholds for antibiotic resistance and virulence factors, number of computational threads, and optional inclusion of geNomad modules for plasmid and virus detection.

BacExplorer is a software developed using Snakemake, complemented by a user-friendly app built with Electron (Fig. 2A). It integrates several tools and databases, which are downloaded via Bioconda (Grüning *et al.* 2018). The software can analyze both raw sequences (FASTQ) and aligned sequences (FASTA) (Fig. 2B). It generates an output consisting of an HTML report and a series of Excel tables that summarize the results from each tool, organized by topic.

### 3.1 Report

**Figure 3.**
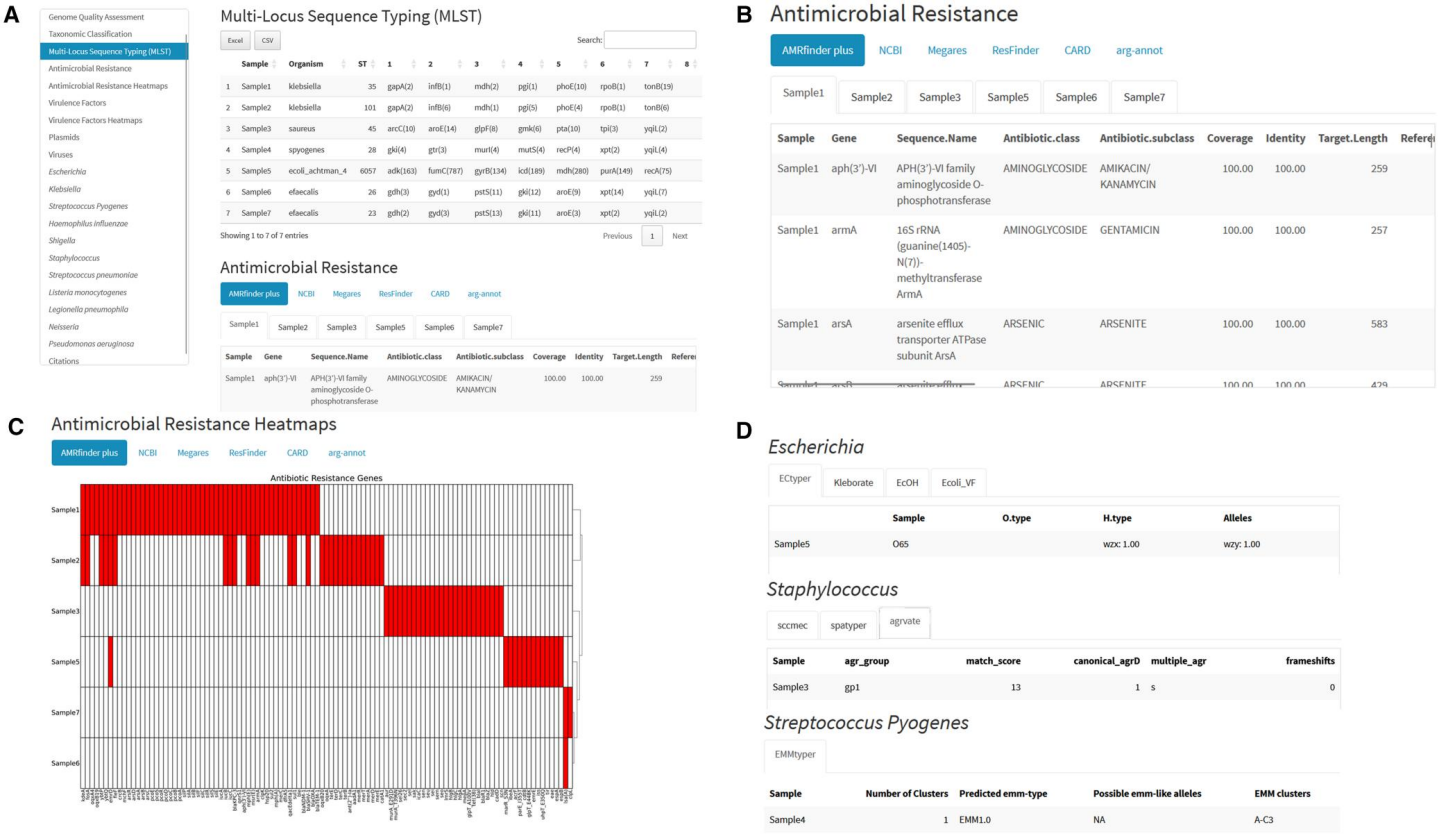
Screen of the BacExplorer HTML Report. A) Multi-locus sequence typing. B) Antimicrobial resistance tables with a look up on AMRfinder+ database. C) Example of antimicrobial resistance heatmap. D) Examples of species/genera-specific analysis for Escherichia, Staphylococcus and *Streptococcus pyogenes*.

BacExplorer generates a flexible and easy to inspect HTML report based on R Markdown. As shown in Fig. 3A, the reports consists of 22 sections containing different analysis types. The report includes also individual sections for bacteria species-specific analysis. The first two sections include raw reads and genome assembled quality control followed by kraken2 taxonomic identification and MLST analysis. Next, the antibiotic resistance section consists of a series of tables, one per sample (Fig. 3B) and per database. The section includes also a series of heatmaps resuming the results of each database (Fig. 3C). The sections that follows are: the virulence, the plasmid and the virus ones. Subsequent, follows the species-specific bacteria section for *Escherichia* spp., *Klebsiella* spp., *S. pyogenes*, *H. influenzae*, *Shigella* spp., *Staphylococcus* spp., *S. pneumoniae*, *Listeria Monocytogenes*, *L. pneumophila*, *Neisseria* spp. and *P. aeruginosa* (Fig. 3D). The report is generated by making use of few R packages such as: dplyr (Wickham *et al.* 2025), stringr (Wickham 2025) for file manipulation, kableExtra (Zhu 2024) to visualize the tables, ComplexHeatmap (Gu *et al.* 2016), and circlize (Gu *et al.* 2014) for generating and visualizing Heatmaps and openxlsx (Schauberger and Walker 2024) generate interactive tables.

### 3.2 Case study

BacExplorer has been tested on several bacterial genomes that are pathogens for humans. Here we report a case study with the analysis of seven genomes (two *Klebsiella pneumonia*, two *Enterococcus faecalis*, one *S. pyogenes*, one *E. coli*, and one *S. aureus*) highlighting the diverse range of analyses our software perform. All the strains have been sequenced at the University of Catania. The DNA was extracted through the QIAGEN QIAamp DNA Mini Kit (Ref.51304, QIAGEN, 40724 Hilden, Germany) and sequenced with the Illumina MiSeq platform according to the manufacturer’s instructions provided in the Illumina DNA Prep.

- *K. pneumoniae* have been identified by MLST, respectively with ST35 for Sample1 and ST101 for Sample2 (Fig. 3A). Regarding AMR analysis, the two *Klebsiella* showed resistance to fosfomycin through *fosA* gene, to gentamicin through *amrA* gene and to quinolones through *oqxA4* with these resistance observable in all the database used by BacExplorer. From AMR Heatmaps it can be notice that the two *Klebsiella* cluster together showing their mutation similarity. Concerning virulence factor it is possible to notice that both the *Klebsiella* present the same 14 virulence genes in VFDB database. For plasmid detection, it is noteworthy to highlight the identification of the plasmid lncHl1B_1_1_pNDM_MAR in both *Klebsiella*. Kleborate identifies the pneumo complex O1ab with the O locus O1/O2v1 and an acquired *bla_KPC3_* gene in both Klebsiella strains. Additionally, the K loci identified are KL22 and KL17, respectively.
- *S.aureus* have been identified by MLST with ST45 (Fig. 3). It showed, in all the AMR databases, the gene *blaZ* that confers resistance to Beta-lactames. In both the virulence factor database we can observe the presence of *sec* genes that produce the enterotoxin C. However, this is the only common gene among the two databases, showing the importance of a comprehensive analysis with the unification of several databases. Concerning genus-specific analysis, agrvate recognizes the *agr* group of gp1 and spatyper gives out t026 as result, while sccmec does not recognize any type because the *S. aureus* is methicillin-sensible.
- *S. pyogenes* have been identified by MLST with ST28 (Fig. 3A). It is annotated using only two AMR databases, Megares and CARD, which identify the gene *lmrP* as conferring resistance to tetracycline due to its role as part of the efflux pump family. For virulence factors, only VFDB provides results for this sample, including *smeZ*, a streptococcal mitogenic exotoxin. In the species-specific analysis, EMMtyper identifies the *S. pyogenes* strain as emm-type EMM1.0, belonging to the EMM cluster A-C3.
- *E. coli* have been identified by MLST with ST6057 (Fig. 3A). It express, both for Megares and for CARD, the *CRP* gene that gives resistance to fluoroquinolones and macrolides since it represses the multidrug efflux pump expression. For *E. coli* virulence factor we can inspect both the “virulence factors” section and the “Escherichia” section, thanks to the use of genera-specific virulence factor finder tool called ecoli_vf. For instance, in both VFDB and ecoli_vf we can found *entB*, an enterobactin. Genera-specific analysis gave us other information, such as the sample serotype recognized as O65 by ECTyper.
- *E. faecalis* have been identified by MLST, respectively, with ST26 for Sample6 and with ST23 for Sample7 (Fig. 3A). Five common AMR genes were identified in both the genomes: macrolide (MLS) *mph(D)*, trimethoprim (*dfrA*), drug and biocide resistance (*efrA* and *efrB*), a multidrug efflux pump (*emeA*), and clindamycin quinupristin-dalfopristin (dalfopristin) [(MLS) *lsaA*]. Notably, the *lsa* gene was detected in both samples across all databases. In terms of virulence gene content, the comparison of both databases evidenced nine core virulence factors associated with biofilm formation (*bopD*), immunomodulation (*cpsA*, *cpsB*), adherence (*ebpA*, *ebpB*, *ebpC*, *srtC*), endocarditis-specific antigen (*efaA*), and surface fibrinogen-binding protein (*fss1*). While, *ace* gene, a collagen adhesin precursor, the *gelE-sprE* interlinked genes, associated with Q/S control of gelatinase and serine-protease expression, and *prgB* gene, which encodes an adhesin that plays a significant role in cellular aggregation and robust biofilm formation, were present only in Sample6. In contrast, VFDB database revealed in Sample7 several other cps genes that code for enzymes involved in capsular polysaccharide synthesis, which possess anti-phagocytic, immune-evasion, and immune-modulation properties. Two plasmids, both belonging to IncP-1 broad-host-range replicon family known for its ability to mobilize and disseminate antibiotic resistance genes, were detected: Sample6 showed rep8b 1 repA(pEJ97p1), belonging to the IncP-1β subfamily, more frequently linked to plasmids carrying AMR genes; Sample7 possesses rep6 1 repA(pS86), belonging to the IncP-1α subfamily, often associated with plasmids carrying genes for heavy metal resistance.

All other information can be inspected in the case study report on our Github page: https://github.com/knowmics-lab/BacExplorer.

### 3.3 Comparison with the existing tools

We compared BacExplorer with seven recently published bacterial genome annotation tools. An overview of their main features and specifications is provided in Table 2, while Table S1, available as supplementary data at *Bioinformatics Advances* online, details the software components and databases utilized by each tool for bacterial genome annotation.

**Table 2.** Feature comparison: BacExplorer vs. existing annotation software.

	BacExplorer	ASA3P	Bacpipe	Bactopia	Campype	Patric	rMAP	Tormes
Quality control	FastQC	FASTQC, FastQ Screen	FastQC	FastQC	FASTQC, MultiQC	FastQC	FASTQC, MultiQC	/
Trimming	TrimGalore	Trimmomatic, Filtlong	TrimGalore	Trimmomatic, BBDuk	Trimmomatic, PRINSEQ	Trim Galore	Trimmomatic	Trimmomatic, Prinseq, Sickle
Taxonomy classification	Kraken2	Kraken, BLAST+ (Silva), MUMmer (ANI)	/	GTDB-tk	Kraken2	Kraken2	/	Kraken2
Alignment	SPAdes	SPAdes, HGAP4, Unicycler	SPAdes	Shovill (Megahit, skesa SPAdes, Velvet), Unicycler	SPAdes	Spades, Canu, Unicycler	Shovil, Megahit	SPAdes, Megahit
Assembly quality	QUAST	/	/	CheckM, QUAST	QUAST	CheckM, Internal quality assesser	QUAST	QUAST
Genome annotation	/	Prokka	Prokka, Barranp, ARAGORN	Prokka	Prokka, DFAST	/	Prokka	Prokka
Contig ordering	/	MeDuSa	/	/	progressiveMauve	progressiveMauve	/	progressiveMauve
Pangenome analysis	/	Roary, FastTreeMP	/	Roary, FastANI, phyloflash	Roary	/	Roary, roary2svg, FastTree	Roary, roary2svg, FastTree
MLST	PubMLST, MLST	BLAST+, PubMLST	MLST	MLST, PubMLST	MLST	/	MLST, PubMLST	MLST, PubMLST
Antibiotic resistance	AMRFinderplus, Abricate	RGI	Blast	AMRFinderplus, Ariba	AMRFinder, Abricate	KMA	AMRFinderplus, Abricate	Abricate, Blast
Virulence	Abricate, VirulenceFinder	BLAST+	Blast	Ariba	Abricate, Blast	KMA	Abricate, Blast	Abricate, Blast
Plasmids	PlasmidFinder, geNomad	/	PlasmidFinder	Ariba	Abricate, Blast	/	Abricate, Blast, PlasmidFinder	PlasmidFinder
Alignment to a reference	/	Bowtie2, PacBio, Minimap2	/	Snippy, BWA, Bedtools, minimap2	Snippy	Bowtie2, Minimap2, Samtools	BWA	/
SNPs call	/	SAMtools, SNPeff	ParSNP	Snippy, vcf-annotator	/		Freebayes, SnpEff	/
Language	Bash, Snakemake, R, Javascript	Groovy (Java)	Python	Nextflow language	Python	NA	Shell	Shell
Input	Fastq/Fasta	Fastq/bam/bax.h5/Fasta/GBK/EMBL/GFF	Fasta/Fastq	Fastq/Fasta	Fastq/Fasta	Fastq/Fasta	Fastq	Fastq/Fasta
Sample type	paired-end single-end	paired-end single-end	paired-end	paired-end single-end	paired-end	paired-end single-end	paired-end	paired-end
Reads	short reads	short reads and long reads	short reads	short reads and long reads	short reads	short reads and long reads	short reads	short reads
Output	Text file, Excel, HTML Report	Text file, JSON files, HTML5 reports	Excel, Text file	JSON/TSV	HTML Report, CSV	HTML Report	HTML	Text file, HTML Report
Software distribution	Docker, Electron	Docker, OpenStack	Docker, Local	Conda, Mamba	Conda, Virtual Machine	Web based	Conda	Conda, mamba, manually
App	Yes	No	Yes	No	No	No	No	No
Last version	v.1—07/2025	v.1.3.0—05/2020	v.1.2.6—10/2019	v.3.2.0—03/2025	v.1.0—02/2020	NA	v.2.1—02/09/2020	v.1.3.0—06/2021

Specifically, the comparison includes:

- **ASA³P** is a pipeline for the assembly and annotation of bacterial isolates. It uses already published tools with fixed parameters designed for reproducibility according to best practice. ASA³P can also receive as input an already assembled genome (contigs or scaffold) and start with annotation. The final stage consists of the aggregation of the results in an HTML output. The last version of the tool (v1.3.0) was released in May 2020, but the last github update results in July 2022.
- **Bacpipe** is a freely available pipeline with a graphical interface. Its analysis can start both with raw and assembled data. The user can decide which tool to activate or deactivate for the analysis and it can adjust the parameters to his needs. Bacpipe outputs are summarized in an excel file, but all the details are reported in folders dedicated to the single tools used. The last release of the tool (v1.2.6) was in October 2019.
- **Bactopia** is a tool for flexible analysis of bacterial Illumina genome sequencing designed as an integrated suite of workflows. It connects open-source bioinformatics software, available from Bioconda, using Nextflow. Its installation is easy thanks to the possibility of installing using not only Bioconda, but also a Docker container or a Singularity container. The tool accepts as input both FASTQ and FASTA and it permits to run several analyses in addition to the basic bactopia pipeline. The last version of the tool is the v.3.2.0 release on the March 2025.
- **Campype** is an open-source workflow for the WGS analysis of paired-end Illumina reads from *C. jejuni* and *E. coli*. Anyway, Campype can analyze any other bacterial genus. The output consists of both HTML and csv report. The tool can be downloaded using conda making the installation easy and avoiding possible environment incompatibility. The latest release of Campype dates back to February 2020 while the last update is in January 2024.
- **PATRIC—**The Pathogen Systems Resource Integration Center is a genomics-focused relational database and bioinformatics resource designed to support scientists in infectious disease research. PATRIC has begun since 2014 to have informatic services usable through its web-service. Among the possible services we have: genome assembly, genome annotation, reconstruction of metabolic models, analyzing SNPs and doing RNAseq experiments. Meanwhile, it also stores more than 250 000 microbial genomes with their metadata permitting the comparison between users’ results and the genomes in the databases. Each user possesses a private workspace where he can upload private data and starts the analysis.
- **rMAP—**The Rapid Microbial Analysis Pipeline—is a one-stop tool that uses WGS data to characterize bacterial resistome. In particular, it was developed for the ESKAPE pathogens group (*S. aureus, P. aeruginosa* and *Klebsiella spp.*). It takes only raw data as input and it was developed using preexisting tools. At the end of the analysis a HTML report is generated. rMAP was developed to help the user with limited bioinformatic knowledge, indeed, it does not require pre-processing of the data or the use of a metadata file. The first (and last) version of the tool was released in 2021, but the last github update was done in December 2023.
- **TORMES** is described as an open-source, user-friendly, command-line pipeline for conducting WGS analysis of bacterial data produced by Illumina platforms. TORMES automates all steps included in a typical WGS analysis permitting the use by non-bioinformaticians. It starts with raw data, but it can also accept already assembled genomes. Its output is a HTML Report created using Rmarkdown with plots easy to understand, it also gives out text files. The current version 1.3.0 has been released in June 2021. The installation instructions have been updated in March 2023.

Table S2, available as supplementary data at *Bioinformatics Advances* online, presents the time required by each tool to analyze FASTA and FASTQ files. For FASTA inputs, BacExplorer and Tormes were the fastest (4-5 minutes), while Bactopia led for FASTQ.

In terms of installation, PATRIC stands out with its fully online platform (https://www.bv-brc.org/). BacExplorer offers the simplest local setup via a downloadable package with a GUI-guided process. Among command line tools, Bactopia, Campype, rMAP, and Tormes are easy to install using Conda.

Several tools presented challenges: rMAP failed due to a MEGAHIT error; Campype had ambiguous input formatting and lacked single-sample support; 5CT encountered a Python error with FASTQ; Bacpipe requires an undocumented graphical interface on Windows and lacks non-root CLI support; Bactopia only accepts zipped files; and ASA3P’s Docker setup omits key instructions, requiring manual intervention. From a usability standpoint, all the state-of-the-art tools that have been compared with BacExplorer operate exclusively via the command line. In contrast, BacExplorer includes step-by-step installation guidance. Through its GUI, our open-source system, allows users to easily upload their raw data folder (or FASTA files) and get as output comprehensive analysis results (yielded as Excel files and HTML reports with interactive heatmaps for streamlined result interpretation). Using these consistent, observable criteria (installation pathway, interface modality, input handling, and report generation), our comparison provides an objective basis for the claim that BacExplorer is easier to use, enabling microbiologists to perform complex bacterial genome analyses independently and without prior computational expertise.

## 4 Discussion

The advent of HTS has revolutionized microbiology, enabling a deeper understanding of the mechanisms underlying well-known bacterial resistances. Next-generation sequencing (NGS) analysis assists microbiologists in identifying and analyzing bacterial strains with unprecedented accuracy. However, comprehensive genome annotation often requires the integration of multiple specialized bioinformatics tools—a process that is both challenging and time-consuming.

While the plethora of bioinformatics tools for bacterial genome assembly, annotation, and visualization offers significant advantages, it also presents notable issues. Each tool comes with its own strengths and limitations, operates in diverse computational environments, and often requires intricate parameter adjustments. This complexity can overwhelm researchers, particularly those without specialized training. Even experienced bioinformaticians may struggle to integrate results from different tools effectively.

Existing tools, though valuable, often lack user-friendly applications critical for use by non-computer scientists. Additionally, many of these tools either lack an intuitive user interface or, like PATRIC, require sample data to be uploaded to external servers. This approach raises privacy concerns, particularly in hospital microbiology laboratories where patient confidentiality is paramount.

To address these challenges and simplify bacterial genome analysis, we developed BacExplorer, a user-friendly tool designed to automate the execution, parsing, and integration of results from various annotation tools. Even though it is not always the fastest, by streamlining the workflow, BacExplorer enhances accessibility to advanced genome annotation for researchers. Its primary goal is to make HTS analysis routine in laboratories, enabling the rapid and accurate examination of bacterial sequences to uncover insights into bacterial resistance mechanisms.

BacExplorer provides a local, streamlined solution for bacterial genome annotation. It enables researchers to incorporate sequencing-derived insights directly into medical reports. By integrating multiple tools and databases with regular monthly updates, BacExplorer ensures comprehensive and up-to-date bacterial annotation. It has already been tested on hundreds of samples at the University of Catania (Bongiorno *et al.* 2023, Bivona *et al.* 2024, Maugeri *et al.* 2025), with ongoing evaluations to further refine its performance.

BacExplorer supports a wide range of analyses for human bacteria, from multi-locus sequence typing (MLST) and taxonomy detection to species-specific investigations of the most threatening pathogens. Its outputs are presented through interactive graphics embedded in an HTML-based consultable report, facilitating the interpretation of complex data. Furthermore, the tool leverages a Snakemake pipeline, enabling seamless integration of future expansions and tools, ensuring that the software remains easily updatable.

## 5 Conclusion

BacExplorer was developed to address the need for rapid analysis and annotation of bacterial genomes obtained through HTS. Designed with microbiologists in mind, its app simplifies bacterial genome analysis, overcoming the bottlenecks typically associated with bioinformatics workflows that often require expert users. With BacExplorer, we aim to make HTS analysis more accessible and feasible for a broader range of laboratories, empowering researchers to harness the full potential of genomic data.

### 5.1 Limitations and future perspective

The next version of BacExplorer will introduce several significant enhancements to expand its capabilities and streamline bacterial genome analysis. These updates include the ability to analyze genomes derived from a combination of short and long reads, offering greater flexibility and accuracy in genome assembly.

## Supplementary Material

vbaf281_Supplementary_Data

## Data Availability

The data underlying this article are available in Github repository bacExplorer https://github.com/knowmics-lab/BacExplorer.
